# Safe Inguinal Hernia Repair Under Local Anesthesia With Multimodal Anesthesia in a High-Risk Patient: A Case Report

**DOI:** 10.7759/cureus.112486

**Published:** 2026-07-11

**Authors:** Koki Miya, Takeshi Kato, Taiga Komatsu

**Affiliations:** 1 Surgery, Honjo Daiichi Hospital, Yurihonjo, JPN; 2 Anesthesiology, Honjo Daiichi Hospital, Yurihonjo, JPN

**Keywords:** case report, dexmedetomidine, inguinal hernia repair, local anesthesia, peripheral nerve block

## Abstract

Inguinal hernia repair under local anesthesia is a minimally invasive procedure and is particularly useful for older patients or those with significant comorbidities. However, local anesthesia alone may provide insufficient analgesia, leading to patient discomfort and intraoperative movement, which can compromise the surgical conditions. We report a case in which multimodal anesthesia enabled the safe surgical management of a high-risk patient.

A 90-year-old man with a right inguinal hernia was referred to our hospital. Laboratory evaluation revealed anemia, and further investigations led to the diagnosis of gastric cancer. The patient subsequently underwent total gastrectomy. Postoperatively, he developed pneumonia, which improved with antibiotic treatment. At 30 days postoperatively, he was scheduled for an inguinal hernia repair. Given his severe emphysema due to long-term smoking and recent history of pneumonia, general anesthesia was considered high-risk. Therefore, the procedure was performed under local anesthesia supplemented with multimodal anesthesia, including dexmedetomidine sedation and ilioinguinal and iliohypogastric nerve blocks. Adequate sedation and analgesia were achieved without respiratory compromise, and the surgery was completed safely under stable operative conditions.

Multimodal anesthesia combined with sedation and nerve blocks can overcome the limitations of local anesthesia alone and facilitate the safe surgical management of high-risk patients. This case highlights the importance of a tailored perioperative strategy and close collaboration between surgeons and anesthesiologists.

## Introduction

Inguinal hernia repair under local anesthesia is less invasive than that under general anesthesia and is considered a useful option for older patients and those with significant comorbidities [1,2]. However, local anesthesia alone may not consistently provide adequate intraoperative analgesia, which can lead to patient discomfort, body movement, and instability of the operative field, thereby interfering with surgical procedures [3].

We herein report the case of a 90-year-old man with a history of postoperative pneumonia following a total gastrectomy and severe emphysema due to long-term smoking. Deterioration of respiratory function under general anesthesia was therefore a concern. By combining dexmedetomidine sedation and nerve blocks with local anesthesia, sufficient sedation and analgesia were achieved, and the surgery was completed safely.

## Case presentation

A 90-year-old man (height, 154.5 cm; weight, 47.4 kg) presented to our hospital with a three-day history of right inguinal bulging. His medical history was significant for severe emphysema (forced expiratory volume in 1 s: 57.8%; smoking history of 20 cigarettes per day for 40 years, ceased 10 years prior).

**Figure 1:**
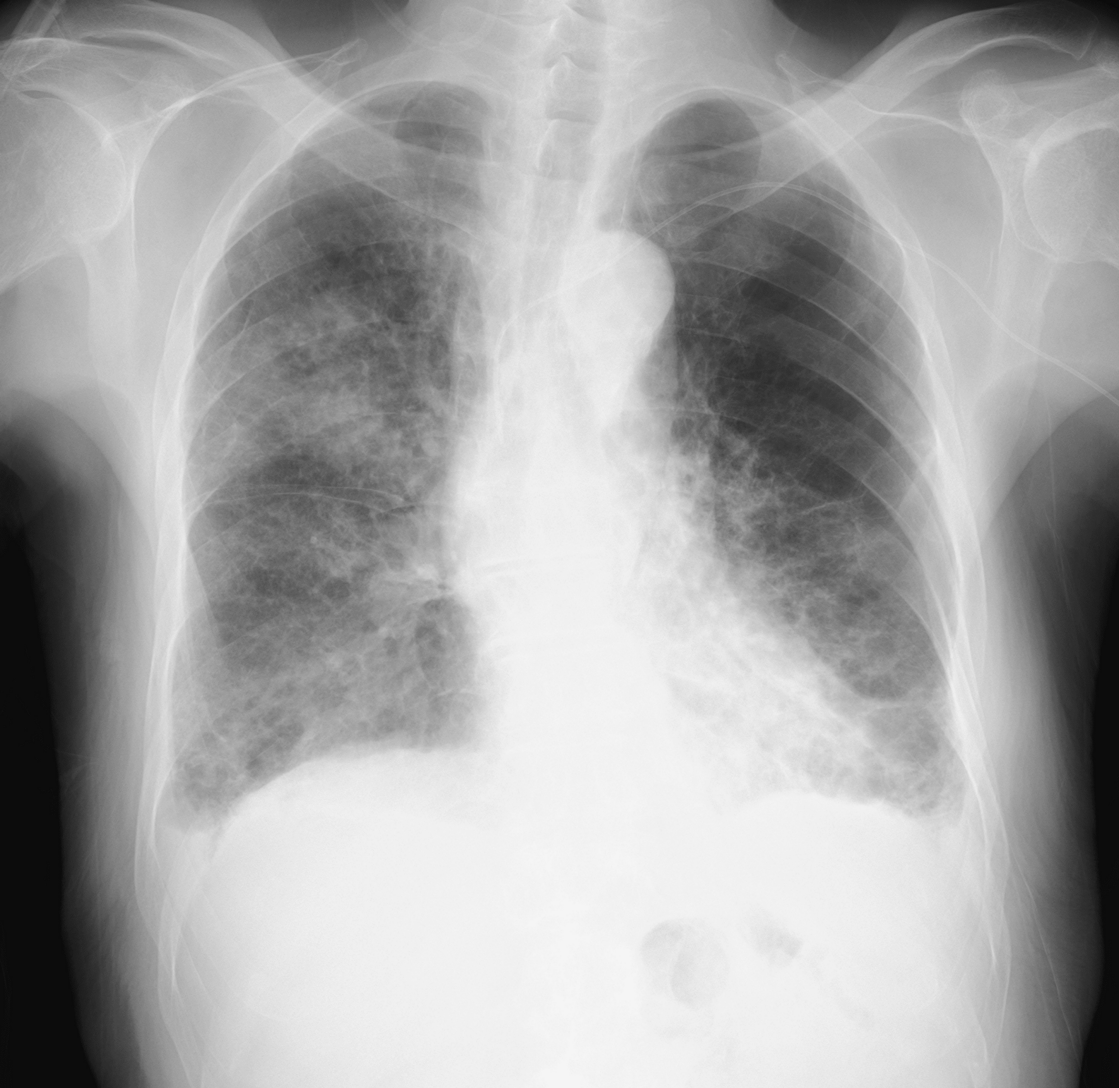
Chest radiograph obtained during postoperative pneumonia after total gastrectomy. The patient developed pneumonia complicated by respiratory failure with CO₂ narcosis, which improved with antibiotic therapy. Considering this recent respiratory complication and underlying severe emphysema, inguinal hernia repair was subsequently performed under local anesthesia with multimodal anesthesia rather than general anesthesia.

A laboratory evaluation for anemia led to a diagnosis of gastric cancer. As no distant metastases were identified, total gastrectomy was performed. Preoperative pulmonary function testing revealed obstructive ventilatory impairment, which was considered to be associated with smoking-related emphysema. Postoperatively, the patient developed pneumonia complicated by respiratory failure with CO₂ narcosis; however, his condition improved after approximately 11 days of antibiotic therapy (Figure 1).

An inguinal hernia repair was planned at 30 days postoperatively. Given the patient’s severe emphysema and recent history of pneumonia, general anesthesia was considered high-risk after consultation with the anesthesiology team. Therefore, surgery under local anesthesia was selected. To overcome the limitations of local anesthesia alone and achieve adequate sedation and analgesia, a multimodal anesthetic strategy combining dexmedetomidine sedation and nerve blocks was planned.

**Figure 2:**
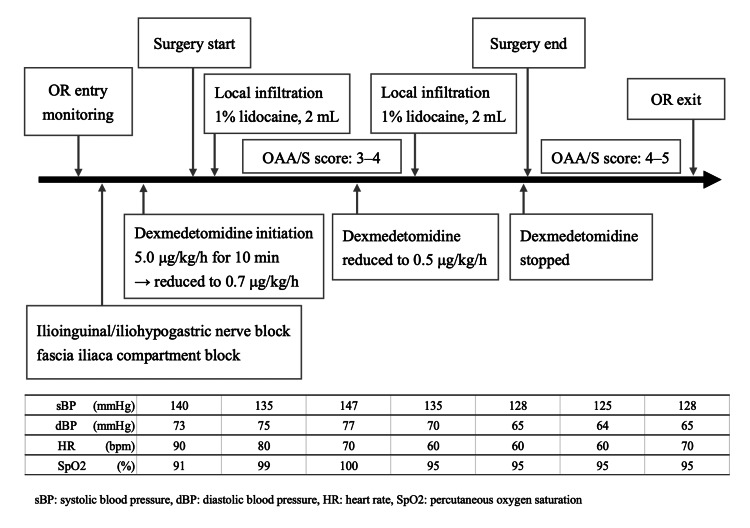
Perioperative timeline of multimodal anesthesia. An ilioinguinal/iliohypogastric nerve block and fascia iliaca compartment block were performed before dexmedetomidine sedation using 20 mL of a mixture of 0.5% ropivacaine and 0.75% mepivacaine for each block. Dexmedetomidine was initiated at 5.0 μg/kg/h for 10 minutes, followed by dose reduction according to the sedation level. Additional local infiltration of 1% lidocaine (2 mL each) was administered during skin incision and manipulation around the internal inguinal ring because of mild pain. Sedation was maintained at an OAA/S score of 3–4 intraoperatively. Representative intraoperative systolic and diastolic blood pressure, heart rate, and oxygen saturation are also shown. These parameters remained stable throughout the procedure. OAA/S: Observer's Assessment of Alertness/Sedation

Due to the history of CO₂ narcosis, low-flow oxygen (3 L/min) was administered via a nasal cannula and subsequently tapered according to the patient's respiratory status. The nerve block was performed prior to the initiation of dexmedetomidine sedation, as the initiation of sedation may complicate patient monitoring and management during the block procedure, particularly in the event of respiratory depression, airway compromise, or over-sedation. An ilioinguinal/iliohypogastric nerve block and a fascia iliaca compartment block were performed using 20 mL of a mixture of 0.5% ropivacaine and 0.75% mepivacaine administered for each block. The total doses of ropivacaine and mepivacaine were 100 mg and 150 mg, respectively. The selection and doses of the local anesthetics were determined by the attending anesthesiologist. Dexmedetomidine was then administered; although the standard loading rate is 6.0 μg/kg/h, a slightly reduced rate of 5.0 μg/kg/h (60 mL/h) was selected for safety considerations and administered for 10 min, followed by a reduction to 0.68 μg/kg/h (8 mL/h). Thereafter, a maintenance infusion of 0.5 μg/kg/h (6 mL/h) was continued according to the level of sedation. During skin incision, the patient reported mild pain, which was managed with additional local infiltration of 2 mL of 1% lidocaine. Mild pain was also noted during manipulation around the internal inguinal ring, for which an additional 2 mL of 1% lidocaine was administered, which was adequately controlled with supplemental local anesthetic administration. Intraoperatively, a right indirect inguinal hernia was confirmed and repaired using the mesh-plug technique, with an operative time of 63 min. Throughout the procedure, sedation was maintained with an Observer's Assessment of Alertness/Sedation score of 3-4 (Figure 2).

Adequate analgesia was maintained throughout the procedure, without frequent supplemental dosing. Dexmedetomidine sedation was safely maintained without respiratory compromise, and the surgery was successfully completed under stable operative conditions. Following postoperative rehabilitation, the patient was discharged on postoperative day 23 without any surgery-related complications.

## Discussion

Inguinal hernia repair under local anesthesia is a minimally invasive and useful approach, particularly in older patients and those with comorbidities [1,2]. Furthermore, in patients with impaired respiratory function, it carries the additional advantage of avoiding perioperative respiratory deterioration and postoperative pulmonary complications associated with general anesthesia. However, local anesthesia alone may not provide sufficient intraoperative analgesia, and patient discomfort and movement can compromise the stability of the surgical field and interfere with the procedure [3].

To address these limitations, multimodal anesthetic strategies that combine sedatives and peripheral nerve blocks have been reported to be effective [3,4]. Suzuki et al. reported the efficacy of combining nitrous oxide with local anesthesia [3]. However, nitrous oxide rapidly diffuses into closed air spaces, increasing gas volume and pressure; therefore, its use is generally avoided in patients with emphysematous lung disease because of the potential risk of bullae expansion or rupture [5]. Furthermore, the use of nitrous oxide has declined in recent years, and intravenous sedatives such as dexmedetomidine have become more commonly used.

Dexmedetomidine is a selective α2-adrenergic receptor agonist with sedative and analgesic properties and is characterized by minimal respiratory depression. Compared with sedatives such as propofol, midazolam, and remimazolam, this respiratory profile makes dexmedetomidine an attractive option for carefully selected patients with severely impaired respiratory function. Although dexmedetomidine may cause bradycardia and hypotension, careful dose titration and close monitoring enabled its safe use in the present patient. It allows the maintenance of a sedated state similar to that of natural sleep and is considered relatively safe, even in older patients and those with impaired respiratory function [6,7]. Additionally, it suppresses intraoperative agitation and body movement, thereby contributing to a stable surgical field. Furthermore, dexmedetomidine has been reported to enhance the analgesic effect of peripheral nerve blocks and prolong their duration when used in combination with local anesthetics, thereby compensating for insufficient analgesia associated with local anesthesia alone [8,9].

Ilioinguinal and iliohypogastric nerve blocks are effective methods for interrupting nociceptive transmission during inguinal surgery and contribute to both intraoperative and postoperative pain control as adjuncts to local anesthesia. Combining these techniques enables the complementary augmentation of analgesia and sedation, which may be insufficient with local anesthesia alone [4]. Although fascia iliaca compartment block is not routinely performed for inguinal hernia repair, it was additionally performed to provide broader regional analgesia should more extensive dissection, including possible femoral canal exploration, become necessary. The combination of ropivacaine and mepivacaine was selected to achieve reliable intraoperative and postoperative analgesia. In this patient, avoiding conversion to deeper sedation or general anesthesia was particularly important because of his extremely limited respiratory reserve. Therefore, an individualized multimodal anesthetic strategy was selected to achieve more reliable analgesia while avoiding general anesthesia. Although no clinical signs of local anesthetic systemic toxicity were observed in the present case, greater attention should have been paid to the cumulative dose of local anesthetics, particularly in this frail, low-body-weight elderly patient.

Spinal anesthesia is another commonly used anesthetic option for older patients. However, the spread of anesthesia increases with age, which may result in a higher-level block [10]. Consequently, circulatory depression and respiratory compromise may be exacerbated, and careful patient selection is required, particularly in those with impaired respiratory function.

In the present case, the patient had severe emphysema and a history of pneumonia and CO₂ narcosis following a total gastrectomy. Therefore, the deterioration of respiratory function associated with airway management under general anesthesia was strongly anticipated. Although avoidance of general anesthesia was considered desirable, local anesthesia alone was expected to be insufficient for adequate analgesia. Spinal anesthesia was also considered suboptimal owing to the potential risk of a high-level block and its impact on respiratory function. Accordingly, after consulting the anesthesiology team, we adopted a strategy that combined local anesthesia with dexmedetomidine sedation and peripheral nerve blocks. As a result, a stable level of sedation was achieved without significant fluctuations in respiratory or hemodynamic parameters during surgery. Adequate analgesia was maintained without the need for frequent additional administration of local anesthetics, and the procedure was safely completed with a stable surgical field. Although epidural anesthesia or low-dose spinal anesthesia with epidural supplementation may also represent reasonable alternatives in selected patients, the optimal anesthetic strategy should be individualized according to the patient's overall clinical condition and respiratory reserve.

This case highlights the importance of collaborative decision-making between surgeons and anesthesiologists in developing flexible, patient-specific anesthetic strategies when local anesthesia alone is insufficient. In selected older patients at high risk for general anesthesia, combining local anesthesia with sedation and peripheral nerve blocks may represent an individualized anesthetic option when careful monitoring and close collaboration between surgeons and anesthesiologists are available.

## Conclusions

Multimodal anesthesia combining dexmedetomidine sedation and peripheral nerve blocks may be an individualized anesthetic option for selected high-risk patients in whom general anesthesia is considered high risk. When local anesthesia alone is expected to provide insufficient analgesia, this strategy may facilitate safe surgery with careful patient selection, close collaboration between surgeons and anesthesiologists, and meticulous perioperative monitoring.
